# Femoral offset aimer use is not associated with improved anatomic accuracy in hamstring ACL reconstruction: A 3D‐CT study

**DOI:** 10.1002/jeo2.70831

**Published:** 2026-06-30

**Authors:** Francisco J. Simón‐Sánchez, Miguel A. Palacios‐Flores, Simone Perelli, Nicola Pizza, Jason Bravo, Àngel Masferrer‐Pino, Joan C. Monllau

**Affiliations:** ^1^ Institut Català de Traumatologia i Medicina del'Esport (ICATME)‐Hospital Universitari Dexeus Universitat Autònoma de Barcelona Barcelona Spain; ^2^ Orthopaedic Surgery Service EsSalud‐Hospital Nacional Edgardo Rebagliati Martins Lima Perú; ^3^ Department of Surgery and Morphologic Science, Orthopaedic Surgery Service, Hospital del Mar Universitat Autònoma de Barcelona Barcelona Spain; ^4^ Orthopaedic Surgery Service Hospital José Casimiro Ulloa Lima Perú

**Keywords:** anterior cruciate ligament reconstruction, Bernard–Hertel quadrant method, femoral offset guide, three‐dimensional computed tomography (3D‐CT), tunnel positioning

## Abstract

**Purpose:**

To assess femoral and tibial tunnel positioning after primary hamstring autograft anterior cruciate ligament (ACL) reconstruction using postoperative three‐dimensional computed tomography (3D‐CT), and to evaluate whether tunnel placement differs with the use of a 7‐mm femoral offset aimer and surgeon experience.

**Methods:**

This retrospective cross‐sectional study included patients aged 18–50 years who underwent Primary single‐bundle hamstring ACL reconstruction between 2022 and 2025 and had postoperative 3D‐CT imaging available. The femoral and tibial tunnel positions were assessed using validated 3D‐CT measurement methods and then classified according to the published anatomic reference ranges. The association of tunnel accuracy with 7‐mm femoral offset aimer use and surgeon experience was analysed. Interobserver reliability was assessed using the intraclass correlation coefficient.

**Results:**

Eighty‐six patients were included (37.2% female; median age 25.5 years). High‐experience surgeons performed the procedures in 39.5% of cases, and a 7‐mm offset aimer was used in 81.4% (*n* = 70). Interobserver reliability was good‐to‐excellent. Mean femoral depth and height were 31.3 ± 4.3% and 29.0 ± 4.5%, respectively, while the mean tibial anteroposterior and mediolateral positions were 40.7 ± 4.3% and 47.0 ± 1.9%. The femoral and tibial tunnels were anatomic in 76.7% and 67.4% of the cases, respectively. Offset aimer use was not associated with femoral anatomic accuracy (*p* = 0.75) but was associated with a more posterior femoral tunnel position (depth 30.7 ± 3.9% vs. 34.5 ± 4.9%; *p* = 0.002). There was no difference in femoral height (*p* = 0.90). Surgeon experience was not associated with anatomic femoral (*p* = 0.46) or tibial (*p* = 0.99) tunnel placement accuracy.

**Conclusion:**

In this retrospective 3D‐CT study, 7‐mm femoral offset aimer use was associated with a more posterior femoral tunnel position but not with improved overall anatomic accuracy. Surgeon experience was not associated with femoral or tibial tunnel accuracy. The clinical relevance of these radiological findings remains uncertain.

**Level of Evidence:**

Level III.

## Graphical abstract

Postoperative 3D‐CT assessment of femoral and tibial tunnel positioning after hamstring ACL reconstruction. The femoral offset aimer was associated with a more posterior femoral tunnel position, but did not improve anatomic accuracy; surgeon experience showed no association with tunnel accuracy.

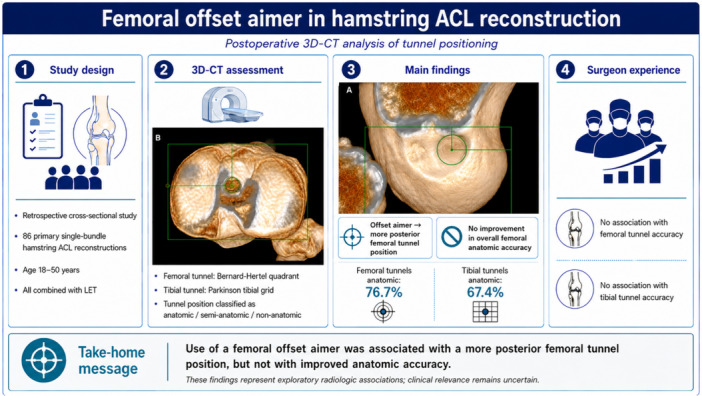

**Abbreviations**
3D‐CT: three‐dimensional computed tomography
ACL: anterior cruciate ligament
AM: anteromedial (transportal technique)
AP: anteroposterior
BPTB: bone–patellar tendon–bone
CI: confidence interval
CT: computed tomography
G: gracilis
ICC: intraclass correlation coefficient
IQR: interquartile range
LET: lateral extra‐articular tenodesis
LFC: lateral femoral condyle
MFC: medial femoral condyle
ML: mediolateral
PACS: picture archiving and communication system
SD: standard deviation
ST: semitendinosus

## INTRODUCTION

Arthroscopic anterior cruciate ligament (ACL) reconstruction is the standard procedure for the surgical treatment of ACL‐related knee instability worldwide [14]. Reported outcomes are generally favourable, with long‐term stability and functional satisfaction seen in approximately 87%–90% of patients, and there were acceptable functional results beyond 7 years [18, 24]. Nevertheless, recent literature continues to emphasize that graft failure, return to sport and revision risk remain clinically relevant concerns after ACL reconstruction [6, 17]. As technical errors remain an important cause of failure, accurate femoral and tibial tunnel placement remains crucial to good graft biomechanics and knee kinematics.

Femoral tunnel malposition is one of the most frequent technical errors. It is commonly characterized by a more anterior and proximal tunnel location. It results in a vertical, non‐anatomic graft orientation [26]. Recent studies have further highlighted the relationship between femoral tunnel malposition, graft orientation and abnormal knee mechanics or the risk of failure after ACL reconstruction [20, 26, 31]. The anteromedial (AM) transportal technique has been proposed to reduce tunnel malposition. It does so by facilitating access to the anatomic femoral footprint, provided portal placement and knee flexion are appropriate [12, 30].

Surgeon experience may influence technical accuracy. A high annual case volume, often defined as more than 35 ACL reconstructions per year, has been associated with more anatomic tunnel placement and fewer technical errors and complications [9]. Adjunctive tools, including computer navigation, intraoperative fluoroscopy, flexible measuring devices and femoral offset guides, have been proposed to improve tunnel placement and reduce technical variability [14, 16]. However, whether such instrumentation improves radiological tunnel accuracy in routine clinical practice remains uncertain. Recent studies in the Journal of Experimental Orthopaedics have also emphasized the relevance of femoral tunnel exit, anatomic femoral tunnel access and hamstring graft position within the femoral socket in ACL reconstruction [1, 8, 10].

Postoperative three‐dimensional computed tomography (3D‐CT) scans provide a detailed assessment of tunnel position and may offer objective feedback for technical evaluation [11]. Validated measurement methods, including the Bernard–Hertel quadrant method for femoral tunnel assessment [13, 32] and the Parkinson tibial grid [21], allow for a standardized evaluation of tunnel position. Although the influence of the surgical technique, instrumentation and surgeon experience on ACL tunnel positioning has been previously investigated, the extent to which a femoral offset aimer affects tunnel position as assessed with 3D‐CT scans in routine clinical practice remains incompletely defined [23].

Therefore, the purpose of this study was to evaluate femoral and tibial tunnel positioning after primary ACL reconstruction with hamstring autograft using postoperative 3D‐CT scans, and to determine whether tunnel placement varied depending on the use of a 7‐mm femoral offset aimer and surgeon experience. It was hypothesized that the use of a 7‐mm femoral offset aimer would be associated with differences in femoral tunnel positioning and that surgeon experience would play a significant role in femoral and tibial tunnel placement accuracy.

## METHODS

This observational, retrospective cross‐sectional study was conducted at a specialized knee surgery centre in Barcelona, Spain. The present study represents a secondary retrospective analysis of patients with available postoperative 3D‐CT imaging from a prior institutional study protocol approved by the local Clinical Research Ethics Committee (approval code: 2022/19‐COT‐DEX). CT acquisition was protocol‐based within the prior approved study and was not prompted by suspected tunnel malposition, postoperative complications, legal concerns or unfavourable clinical evolution.

Anonymized data were extracted from electronic medical records and postoperative imaging. That data were handled confidentially in accordance with the Declaration of Helsinki and Good Clinical Practice standards.

### Participants

Patients were retrospectively identified from the prior ethics‐approved institutional 3D‐CT study cohort. The source population for the present analysis consisted of patients who had undergone postoperative 3D‐CT imaging after ACL reconstruction within that protocol. Predefined inclusion and exclusion criteria were then applied to define the final analytical cohort.

Eligible patients were those with available postoperative 3D‐CT imaging and complete operative documentation regarding the tunnel creation, graft type, operating surgeon, as well as the use or non‐use of a 7‐mm femoral offset aimer. Cases were included in the offset aimer analysis only when operative documentation clearly allowed classification as guide use or non‐use. Cases with incomplete or ambiguous operative documentation regarding offset aimer use were excluded.

**Inclusion criteria**

- Age 18–50 years.
- Primary single‐bundle ACL reconstruction with hamstring autograft (semitendinosus [ST] and gracilis [G]) combined with Lateral extra‐articular tenodesis (LET), reflecting the characteristics of the study cohort.
- Postoperative 3D‐CT is suitable for 3D reconstruction.

**Exclusion criteria**

- Revision ACL reconstruction.
- Use of other grafts (bone–patellar tendon–bone [BPTB], quadriceps tendon, rectus femoris or allograft).
- Incomplete CT studies, insufficient resolution or the inability to process images for 3D reconstruction.
- Incomplete operative documentation.
- Degenerative or inflammatory pathology (Kellgren–Lawrence Grade III–IV osteoarthritis, rheumatoid arthritis, etc.).

Because this was a retrospective secondary analysis of patients from a prior ethics‐approved 3D‐CT study protocol, the available sample was determined by the number of eligible patients in that source study. A minimum analytical target of 80 patients had been set for the radiological assessment of tunnel‐positioning parameters (power 80%, *α* = 0.05). However, that target was not designed to provide adequate power for subgroup or interaction analyses. Malpositioning was defined according to previously published anatomic reference ranges for the femoral and tibial tunnel location. Because clinical outcomes were not assessed, the study was not designed to determine a clinically relevant threshold for tunnel malpositioning.

### Surgical technique

All ACL reconstructions were performed by six surgeons within the knee unit who used an anatomic single‐bundle technique. A hamstring autograft was used in all cases. The graft consisted of ST and G tendons prepared as a four‐strand ST‐G graft. The femoral tunnel was created using the AM transportal technique with cortical suspensory fixation. The tibial tunnel was created using an outside‐in technique with single fixation (interference screw) or double fixation (interference screw plus cortical suspensory fixation).

When used, the 7‐mm femoral offset aimer was applied through the AM portal in line with the surgeon's standard technique. However, because of the retrospective design, detailed information regarding the exact intraoperative knee flexion angle during femoral drilling, portal placement, guide orientation and subtle variations in guide handling was not consistently available for all cases.

All patients underwent combined LET. That said, LET positioning was not a primary outcome of the present study. LET was performed after the creation of the ACL femoral and tibial tunnels.

### CT acquisition and image analysis

Postoperative CT scans were acquired approximately 2 weeks after surgery using a 64‐slice multidetector helical CT scanner (GE). The images were stored in the institutional picture archiving and communication system (PACS) and analysed on a dedicated workstation (Carestream Vue Motion, v12.2.5.0; Carestream Health). Linear and angular tools and window‐level adjustments were used to optimize visualization.

All measurements were performed independently by two orthopaedic surgeons trained in the musculoskeletal imaging assessment of ACL tunnel position. Both observers were blinded to surgeon experience and offset aimer use at the time of measurement. Interobserver reliability was assessed using the Intraclass correlation coefficient (ICC). A two‐way random‐effects model with absolute agreement was used to evaluate agreement between the two independent observers. ICC values were interpreted according to Koo et al. Values below 0.50 were considered poor, 0.50–0.75 moderate, 0.75–0.90 good and above 0.90 excellent reliability [15].

### Variables

Demographic and surgical variables were obtained from electronic medical records. Information regarding the use or non‐use of the 7‐mm femoral offset aimer was taken from operative records. Cases were included in the offset aimer analysis only when operative documentation clearly established whether the guide had been used or not. Surgeon experience was defined conditionally on the annual ACL reconstruction case volume. Surgeons performing more than 35 ACL reconstructions per year were classified as high‐experience surgeons, whereas those performing 35 or fewer ACL reconstructions per year were classified as low‐experience surgeons.^9^

### Outcome measures

#### Femoral tunnel measurements

The 3D distal femur model was aligned in a strict lateral view by superimposing the medial femoral condyle (MFC) and lateral femoral condyle (LFC). Then, the MFC was removed with a sagittal cut at the highest point of the intercondylar notch, and the remaining LFC was returned to a strict lateral position using the initial reference [13]. The Bernard–Hertel quadrant method was applied to the medial wall of the LFC [4]. Standardized boundaries were used to minimize variability [27].

**Figure 1:**
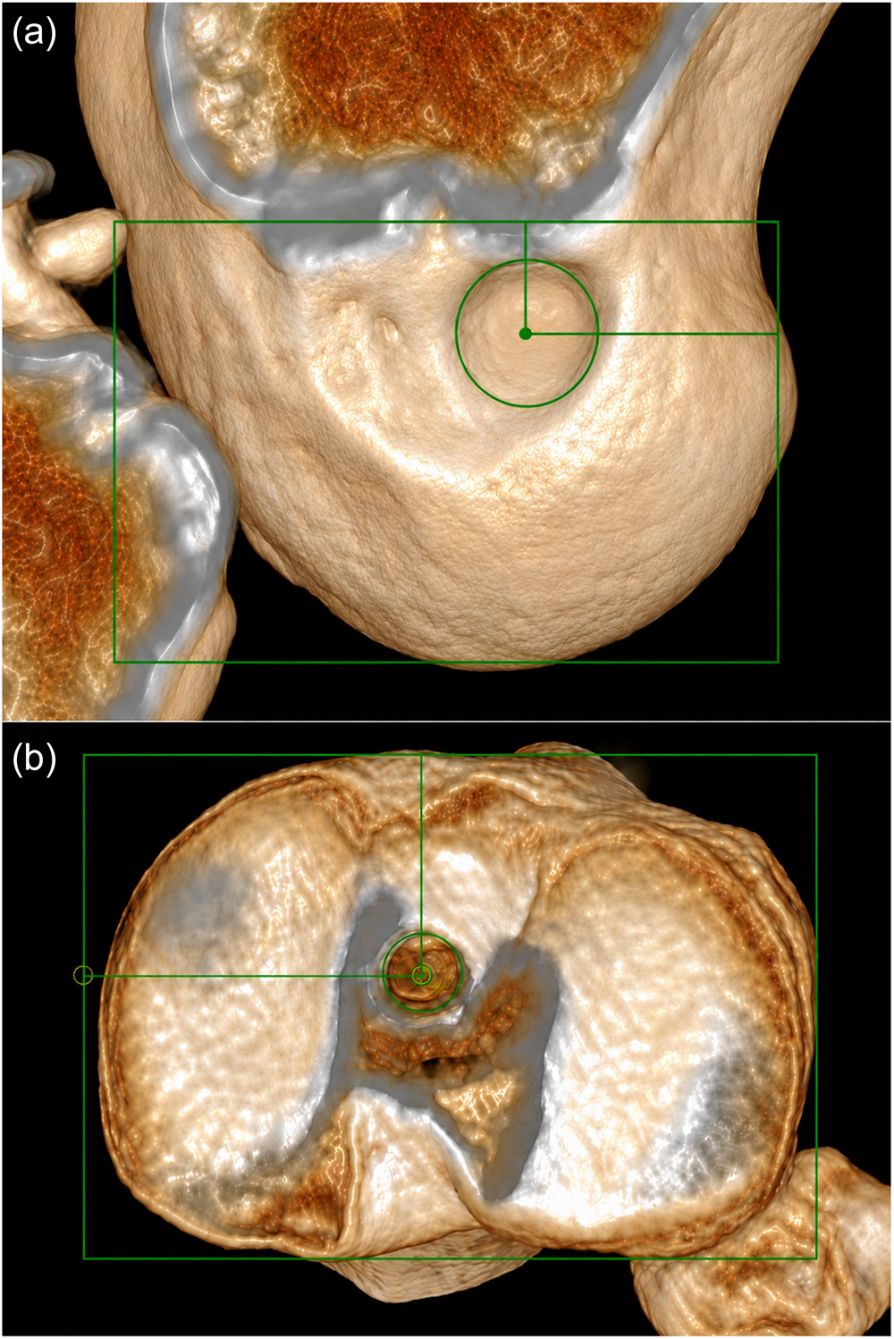
Three‐dimensional computed tomography measurement methods. (a) Femoral tunnel position was assessed using the Bernard–Hertel quadrant method, with depth and height expressed as percentages of the corresponding axes. (b) Tibial tunnel position was assessed using a standardized tibial grid, with anteroposterior and mediolateral positions expressed as percentages of the corresponding dimensions.

The femoral tunnel position was defined as the centroid of the femoral tunnel aperture. Depth (deep–shallow axis) and height (superior–inferior axis) were expressed as percentages of the corresponding axis lengths (Figure 1a). The femoral tunnel position was assessed using the Bernard–Hertel quadrant method because it is a validated and widely used method for the postoperative assessment of femoral tunnel location and permits comparisons with previous 3D‐CT studies. The femoral anatomic reference depth value was 28.6% (95% confidence interval [CI] 23.5–37.3), and the height value was 34.5% (95% CI 28.4–42.6) [19]. A tunnel was classified as anatomic when both parameters were within the corresponding reference ranges, semi‐anatomic when only one parameter was within range, and non‐anatomic when neither parameter was within range [2, 25].

### Tibial tunnel measurements

The 3D proximal tibia model was oriented in a true lateral view from the medial aspect with both femoral condyles superimposed on each other. Then, the patella and the femur were removed. The distal tibia was cut at the maximal proximal width using a cut parallel to the slope of the medial tibial plateau (Amis and Jakob line) [3]. The remaining proximal fragment was viewed from directly above, and a rectangular grid was aligned with the anterior, posterior, medial and lateral cortices [5, 21, 29].

The distances from the anterior and medial borders to the tibial tunnel centre were measured in millimetres, and the anteroposterior (AP) and mediolateral (ML) positions were expressed as percentages (Figure 1b). The tibial anatomic reference values were AP 42.3% (95% CI 38.5–45.5) and ML 48.0% (95% CI 44.7–51.3) [19, 21]. The femoral and tibial tunnel positions were classified as anatomic, semi‐anatomic or non‐anatomic according to previously published reference ranges. For the purposes of the present analysis, malpositioning was defined radiologically as tunnel placement outside the corresponding anatomic reference range [2, 25].

### Statistical analysis

Analyses were performed using RStudio (Posit Software). Continuous variables were summarized using appropriate measures of central tendency and dispersion, and categorical variables were expressed as frequencies and percentages. For bivariate analyses, femoral and tibial accuracy were dichotomized as anatomic versus semi‐/non‐anatomic. Associations between offset aimer use, surgeon experience and categorical tunnel accuracy outcomes were assessed using the *χ* ^2^ or Fisher's exact tests, as appropriate. Continuous tunnel positioning parameters were compared between groups using the Student's *t* test or the Mann–Whitney *U* test, depending on the data distribution.

Given the limited number of patients in the non‐offset aimer group and the imbalance between comparison groups, analyses in consideration of offset aimer use and surgeon experience were considered exploratory. Multivariable regression was not performed because of the limited sample size, group imbalance and the risk of model overfitting and unstable estimates. No formal correction for multiple comparisons was applied. Therefore, *p* values should be interpreted cautiously, particularly for secondary and exploratory comparisons. Statistical significance was set at *p* < 0.05.

## RESULTS

### Study population and reliability

**Figure 2:**
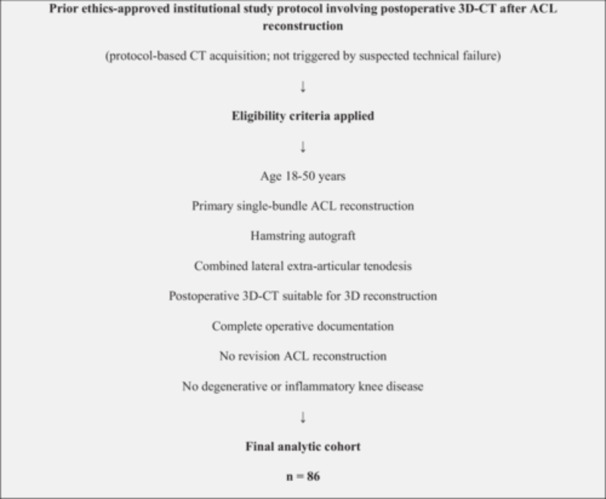
Cohort selection diagram showing the derivation of the final analytic cohort from the prior ethics‐approved institutional 3D‐CT study cohort. 3D‐CT, three‐dimensional computed tomography; ACL, anterior cruciate ligament.

The cohort selection process is summarized in Figure 2. Patients were identified from the prior ethics‐approved institutional 3D‐CT study cohort. After the application of the predefined eligibility criteria, 86 patients were included in the final analysis. Interobserver reliability was assessed using a two‐way random‐effects ICC model with absolute agreement. Reliability was good to excellent for all femoral and tibial measurements in accordance with the criteria proposed by Koo et al. (Table 1).

**Table 1:** Interobserver reliability of analysed measurements.

Measurement	*n*	ICC	95% CI	*p* Value	Reliability (15)
Femoral height (%)	86	0.89	0.84–0.92	<0.001	Good
Femoral depth (%)	86	0.84	0.78–0.88	<0.001	Good
Tibia AP (%)	86	0.82	0.75–0.87	<0.001	Good
Tibia ML (%)	86	0.77	0.68–0.84	<0.001	Good

*Note*: Study: Femoral offset aimer use is not associated with improved anatomic accuracy in hamstring ACL reconstruction: a 3D‐CT study.

Abbreviations: AP, anteroposterior; CI, confidence interval; ICC, intraclass correlation coefficient; ML, mediolateral.

### Baseline characteristics

Baseline demographic, surgical and radiologic characteristics are summarized in Table 2. Detailed numerical values are presented in the table.

**Table 2:** Participant characteristics (*n* = 86).

Characteristic	Value
Age (years), median (IQR)^a^	25.5 (20–33)
Female sex, *n* (%)	32 (37.2)
High‐experience surgeon, *n* (%)	34 (39.5)
Use of 7‐mm femoral offset guide, *n* (%)	70 (81.4)
Right knee, *n* (%)	46 (53.5)
Tibial tunnel diameter (mm), mean ± SD^b^	9.6 ± 0.6
Tibial interference screw diameter (mm), mean ± SD^b^	10.1 ± 0.7
Tibial interference screw length (mm), median (IQR)^a^	25 (20–25)
Femoral depth (%), mean ± SD^b^	31.3 ± 4.3
Femoral height (%), mean ± SD^b^	29.0 ± 4.5
Tibia AP (%), mean ± SD^b^	40.7 ± 4.3
Tibia ML (%), mean ± SD^b^	47.0 ± 1.9
Femoral accuracy: Anatomic/semi‐anatomic/non‐anatomic, *n* (%)	66 (76.7)/19 (22.1)/1 (1.2)
Tibial accuracy: Anatomic/semi‐anatomic/non‐anatomic, *n* (%)	58 (67.4)/24 (27.9)/4 (4.7)

Abbreviations: AP, anteroposterior; IQR, interquartile range; ML, mediolateral; SD, standard deviation.

^a^ Normally distributed variables reported as mean ± SD.

^b^ Non‐normally distributed variables reported as median (IQR).

### Tunnel positioning

Femoral and tibial tunnel positioning measurements and accuracy classifications are shown in Table 2. Overall, femoral tunnel placement was classified as anatomic in 76.7% of cases, and tibial tunnel placement was classified as anatomic in 67.4% of cases.

### Associations with accuracy and positioning parameters

Surgeon experience was not associated with anatomic femoral or tibial placement accuracy. The use of the 7‐mm offset aimer was not associated with femoral anatomic accuracy (Table 3). In an exploratory subgroup analysis limited to cases in which the 7‐mm femoral offset aimer was used, anatomic femoral tunnel placement accuracy did not differ significantly between high‐ and low‐experience surgeons (Table 4). This finding should be interpreted cautiously because of the limited sample size and the uneven distribution of offset aimer use across surgeon‐experience groups.

**Table 3:** Bivariate analysis of surgeon experience, offset guide use and accuracy (anatomic vs. semi‐/non‐anatomic).

Characteristic	Femoral anatomic *n* (%)	Femoral semi‐/non *n* (%)	*p* Value	Tibial anatomic *n* (%)	Tibial semi‐/non *n* (%)	*p* Value
Surgeon experience: High	28 (82.4)	6 (17.6)	0.46	23 (67.6)	11 (32.4)	0.99
Surgeon experience: Low	38 (73.1)	14 (26.9)		35 (67.3)	17 (32.7)	
7‐mm offset guide: Yes	53 (75.7)	17 (24.3)	0.75			
7‐mm offset guide: No	13 (81.3)	3 (18.7)				

*Note*: Tests: *χ* ^2^ or Fisher's exact test, as appropriate.

**Table 4:** Femoral accuracy according to surgeon experience among cases using a 7‐mm femoral offset guide (*n* = 70).

Surgeon experience	Anatomic *n* (%)	Semi‐/Non‐anatomic *n* (%)	*p* Value
High experience	15 (83.3)	3 (16.7)	0.50
Low experience	38 (73.1)	14 (26.9)	

*Note*: Test: Fisher's exact test.

The use of the offset aimer was associated with a lower femoral depth value. It reflects a more posterior femoral tunnel position, with no difference in femoral height. Surgeon experience did not influence individual positioning parameters (Table 5).

**Table 5:** Femoral and tibial accuracy parameters depending on guide use and surgeon experience.

Parameter	Offset guide: Yes (mean ± SD)	Offset guide: No (mean ± SD)	*p* Value	Experience: High (mean ± SD)	Experience: Low (mean ± SD)	*p* Value
Femoral depth (%)	30.7 ± 3.9	34.5 ± 4.9	0.002	32.2 ± 4.9	30.8 ± 3.9	0.15
Femoral height (%)	29.0 ± 4.7	29.1 ± 3.6	0.90	28.9 ± 4.0	29.1 ± 4.9	0.70
Tibial AP (%)				40.9 ± 3.8	40.6 ± 4.7	0.70
Tibial ML (%)				46.8 ± 1.9	47.3 ± 2.0	0.30

*Note*: Test: Student's *t* test for all comparisons.

Abbreviations: AP, anteroposterior; ML, mediolateral; SD, standard deviation

## DISCUSSION

The principal finding of this study was that use of a 7‐mm femoral offset aimer during primary single‐bundle hamstring ACL reconstruction was associated with a more posterior femoral tunnel aperture in postoperative 3D‐CT scans. However, offset aimer use was not associated with improved femoral anatomic accuracy. Surgeon experience, dichotomized according to annual ACL reconstruction volume, was not associated with femoral or tibial tunnel accuracy in this cohort. These findings should be interpreted cautiously, given the retrospective design, the non‐randomized use of the offset aimer and the imbalance between comparison groups.

Although previous studies have suggested that femoral offset guides may support reproducible femoral tunnel placement, the present data do not demonstrate that a 7‐mm offset aimer improves anatomic accuracy or compensates for differences in surgeon experience. The subgroup analysis according to surgeon experience was exploratory and limited by the uneven distribution of offset aimer use. Therefore, no conclusion can be drawn regarding whether the offset aimer reduces experience‐related variability.

The observed association between offset aimer use and femoral tunnel depth was limited to the deep–shallow axis. No corresponding difference was observed in femoral height, and anatomic femoral tunnel placement accuracy did not differ between offset aimer and non‐offset aimer groups. Therefore, the finding demonstrates that the use of the offset guide was associated with a different position of the femoral tunnel in the radiological/3D‐CT image, specifically a more posterior one. Stated another way, a difference in the position of the femoral tunnel (more posterior) was seen, but that difference does not necessarily mean that the reconstruction is anatomically better.

The exploratory subgroup analysis among cases performed with the offset aimer did not show a significant difference in femoral anatomic accuracy between high‐ and low‐experience surgeons. However, this analysis was limited by the small sample size and the absence of low‐experience surgeons in the non‐offset aimer group. As a result, the present study cannot evaluate whether offset aimer use modifies the relationship between surgeon experience and femoral tunnel accuracy.

Anatomic femoral tunnel placement accuracy in this series (76.7%) is consistent with previously reported rates using 3D‐CT scans and the Bernard–Hertel quadrant method [2, 7, 25, 28]. Tibial tunnel placement accuracy (67.4%) was slightly higher than values reported in earlier studies using the Parkinson grid [2, 21, 22]. This discrepancy may reflect differences in surgical technique, imaging protocols or reference thresholds rather than superior tibial tunnel placement. Recent studies included in the current reference list also emphasize that tunnel position, graft orientation and surgeon‐related technical factors remain relevant when evaluating ACL reconstruction quality and the risk of failure [9, 26, 31]. Notably, neither surgeon experience nor offset aimer use improved anatomic placement accuracy in the present cohort. This supports the interpretation that the observed difference in femoral depth should be regarded as a limited radiological association rather than evidence of clinically meaningful improvement.

Postoperative 3D‐CT makes for a reproducible radiological assessment of tunnel positioning and may be useful for technical evaluation [11]. However, the present study did not assess knee stability, graft failure, return‐to‐sport, patient‐reported outcomes or the long‐term clinical results. Therefore, the clinical relevance of the observed difference in femoral tunnel depth cannot be determined from these data.

The present findings should be interpreted as exploratory radiological associations rather than evidence of a causal effect of offset aimer use or surgeon experience on tunnel accuracy. The observed difference in femoral depth did not translate into improved anatomic placement accuracy, and its clinical relevance remains uncertain in the absence of clinical, functional or long‐term outcome data.

## LIMITATIONS

This study has several limitations that should be considered when interpreting the findings. First, the retrospective observational design precludes causal inference. This was a selected retrospective cohort derived from a prior ethics‐approved institutional 3D‐CT study protocol rather than from all ACL reconstructions performed during the study period. Therefore, the cohort may not represent the overall ACL reconstruction population. However, CT acquisition was protocol‐based within the prior approved study and was not triggered by suspected tunnel malposition, postoperative complications, legal concerns or unfavourable clinical evolution, which reduces the likelihood of indication bias related to selective imaging of suspected technical failures. Because the inclusion required postoperative 3D‐CT imaging and complete operative documentation, selection bias cannot be excluded.

The use of the 7‐mm femoral offset aimer was not randomized and was unevenly distributed across surgeons and experience levels. Therefore, offset aimer use was not independent of surgeon preference or surgical practice, which limits the ability to isolate the independent effect of the instrument. Technical factors that may influence femoral tunnel position include the exact knee flexion angle during drilling, portal placement, guide orientation and individual variations in guide handling. They could not be systematically assessed because of the retrospective study design.

Although a minimum sample size was estimated for the overall analysis of radiological tunnel positioning parameters, the study was not powered for subgroup analyses or for testing interactions between offset aimer use and surgeon experience. Only 16 patients underwent surgery without the use of the offset aimer, which substantially limits the precision of between‐group comparisons, increases the risk of Type II error and precludes conclusions regarding small clinically relevant differences. No multivariable analysis was performed to adjust for potential confounders such as the individual surgeon, surgical technique, LET technique, case complexity or temporal changes in practice. Because several exploratory comparisons were performed without formal correction for multiple testing, isolated statistically significant findings should be interpreted with caution.

Surgeon experience was dichotomized rather than analysed as a continuous variable or across multiple experience strata. This approach may have reduced sensitivity to detect more subtle associations between surgeon case volume, learning curves and tunnel positioning accuracy. The potential influence of senior supervision during procedures performed by low‐experience surgeons could not be formally assessed. This may have reduced measurable differences between surgeon‐experience groups and should be considered when interpreting the absence of an association between experience and tunnel accuracy.

The classification of femoral tunnel accuracy was based on published Bernard–Hertel quadrant reference ranges. Although this approach permits comparisons with previous studies, the reference intervals are relatively broad and may not capture more precise CT‐based anatomic parameters like the distance from the posterior wall of the LFC to the posterior edge of the tunnel. Therefore, some tunnels classified as anatomic according to quadrant‐based reference ranges may still differ from more specific CT‐based anatomic targets.

All patients underwent combined LET using more than one technique. Because LET was performed after creation of the ACL femoral and tibial tunnels, a direct technical effect of LET on ACL tunnel positioning is unlikely. Nevertheless, the inclusion of only combined ACL reconstruction and LET procedures may limit the generalizability of the findings to isolated ACL reconstruction.

One final limitation is that the clinical, functional and biomechanical outcomes were not assessed. Therefore, the present study cannot determine the clinical threshold at which deviations in tunnel position become relevant for knee stability, graft survival or patient‐reported outcomes, even though tunnel malpositioning was defined according to published anatomic reference ranges. Prospective studies incorporating balanced comparison groups, multivariable adjustment, patient‐reported outcomes and longer‐term follow‐ups are needed to establish whether these technical differences translate into improved clinical results.

## CONCLUSIONS

In this retrospective 3D‐CT study, use of a 7‐mm femoral offset aimer was associated with a more posterior femoral tunnel position but was not associated with improved anatomic placement accuracy. Surgeon experience was not associated with femoral or tibial tunnel accuracy in this cohort. Given the retrospective design, group imbalance and the absence of clinical outcome data, these findings should be interpreted as exploratory radiological associations. Larger prospective studies with balanced comparison groups, multivariable adjustment and clinical outcomes are required to clarify the role of adjunctive instrumentation and surgeon experience in ACL tunnel positioning.

## AUTHOR CONTRIBUTIONS

**Francisco J. Simón‐Sánchez**: Principal investigator; responsible for study design; data collection and manuscript writing; served as one of the surgeons. **Simone Perelli**: Conceived the study idea; contributed to study design and manuscript writing; served as one of the surgeons. **Miguel A. Palacios‐Flores**: Collaborated in developing the study idea; performed measurements for all patients; reviewed the manuscript; performed statistical analyses. **Jason Bravo**: Contributed to data collection; performed measurements for all patients. **Nicola Pizza**: Served as one of the principal surgeons; contributed to data collection. **Àngel Masferrer‐Pino**: Designed the methodology; reviewed the manuscript. **Joan C. Monllau**: Supervised the study; reviewed the manuscript.

## FUNDING INFORMATION

The authors have no funding to report.

## CONFLICT OF INTEREST STATEMENT

The authors declare no conflicts of interest.

## ETHICS STATEMENT

The source cohort was derived from a prior institutional postoperative 3D‐CT study protocol approved by the local Clinical Research Ethics Committee (approval code: 2022/19‐COT‐DEX). The present secondary retrospective analysis used anonymized data from that approved protocol and electronic medical records.

## Data Availability

The datasets generated during the current study are available from the corresponding author on reasonable request. The data are not publicly available due to privacy or ethical restrictions.
